# Association Between ^18^F-FDG PET Activity and HER2 Status in Breast Cancer Brain Metastases

**DOI:** 10.1007/s13139-024-00843-8

**Published:** 2024-02-01

**Authors:** Jonathan R. Young, Julie A. Ressler, Joanne E. Mortimer, Daniel Schmolze, Mariko Fitzgibbons, Bihong T. Chen

**Affiliations:** 1 Department of Radiology, Division of Neuroradiology, City of Hope Comprehensive Cancer Center, 1500 E. Duarte Rd., Duarte, 91010 CA USA; 2 Department of Medical Oncology and Therapeutics Research, City of Hope Comprehensive Cancer Center, 1500 E. Duarte Rd., Duarte, 91010 CA USA; 3 Department of Pathology, City of Hope Comprehensive Cancer Center, 1500 E. Duarte Rd, Duarte, 91010 CA USA

**Keywords:** Brain metastasis, Breast cancer, Positron emission tomography, Maximum standardized uptake

## Abstract

**Purpose:**

The objective of this study was to evaluate whether uptake on ^18^F-fluorodeoxyglucose (^18^F-FDG) PET could help differentiate HER2-positive from HER2-negative breast cancer brain metastases.

**Methods:**

In this retrospective, cross-sectional study of a cohort of 14 histologically proven breast cancer brain metastases, we analyzed both preoperative ^18^F-FDG PET/CT and HER2 status of the resected/biopsied brain specimens. The maximum standardized uptake values (SUVmax) of the lesions were normalized to contralateral normal white matter and compared using Mann–Whitney *U* tests.

**Results:**

The study cohort was comprised of 12 women with breast cancer with a mean age of 59 years (range: 43–76 years) with a total of 14 distinct brain metastatic lesions. The SUVmax ratio of HER2-positive breast cancer brain metastases was significantly greater than that of HER2-negative lesions (3.98 vs 1.79, *U* = 38.00, *p* = 0.008).

**Conclusion:**

The SUVmax ratio may help to identify the HER2 status of breast cancer brain metastases, if validated prospectively.

## Introduction

Approximately 30–50% of patients with metastatic breast cancer develop brain metastases [1]. Patients with human epidermal growth factor 2 (HER2)-positive breast cancer have an even greater risk of developing brain metastases when compared to patients with HER2-negative breast cancer [2]. Among women with metastatic HER2 positive breast cancer, 19% have central nervous system (CNS) metastases at diagnosis, and greater than 50% will develop brain metastases over the course of their disease [3–5]. Historically, HER2-positive breast cancers have been associated with a more aggressive clinical course and poorer cancer-specific outcomes in comparison to HER2-negative breast cancers [6, 7]. However, HER2-directed therapies, such as trastuzumab, pertuzumab, and trastuzumab deruxtecan, have been shown to improve survival outcomes for patients at all stages of HER2-positive breast cancer [1, 6, 8–10].

Because of tumor heterogeneity and the influence of systemic therapy, HER2 status in a patient can change over the course of time. Multiple groups have reported the presence of HER2 status discordance between the primary breast cancer and metastatic sites, such as within the brain [11–14]. Prior studies have shown that there is discordance between the HER2 status of the brain metastasis and the primary breast cancer in 10–15% of patients [14, 15]. Because repeated biopsies of brain lesions are neither feasible nor practical, a non-invasive method of determining candidacy for HER2-directed therapies is needed.

Several studies have shown that HER2 overexpression promotes glucose uptake and metabolism [16, 17]. Kim et al. evaluated the ^18^F-FDG PET uptake of primary breast cancers and found that HER2-positive breast cancers exhibited more avid ^18^F-FDG uptake than HER2-negative breast cancers [18]. The goal of this proof-of-concept, pilot study was to investigate whether ^18^F-FDG PET uptake could help differentiate HER2-positive from HER2-negative breast cancer brain metastases.

## Materials and Methods

### Patients

We queried our institution’s pathology database to identify all histologically proven cases of breast cancer brain metastases from 2018 to 2022. Fifty-seven histologically proven lesions were identified in this query. Eligibility criteria included patients with a histologically confirmed breast cancer brain metastasis and preoperative ^18^F-FDG PET/CT imaging of the brain. Forty-three lesions were excluded due to lack of a preoperative ^18^F-FDG PET/CT imaging of the brain, resulting in a study cohort of 14 histologically proven lesions. The cohort of 14 histologically proven lesions corresponded to a total of 12 patients, all of whom were women. Two patients each had two HER2-positive lesions. Seven of the 14 brain metastases (50%) were diagnosed at resection, while the remaining 7 brain metastases (50%) were diagnosed at biopsy. For all 14 lesions, HER2 analyses were performed on the resected/biopsied brain specimens. ^18^F-FDG PET/CT imaging for each of the lesions was acquired prior to treatment. This cross-sectional study was approved by the Institutional Review Board; informed consent was waived due to the retrospective nature of this study.

### Brain 18F-FDG PET-CT Acquisition

After fasting for a minimum of 8 h and resting for 10 min in a quiet room, patients received an intravenous dose of approximately 5 MBq/kg of ^18^F-FDG, followed by a 60-min uptake period. All patients had a serum glucose level less than 120 mg/dL at the time of injection. All ^18^F-FDG PET-CT examinations were performed on the Discovery STE scanner (GE Healthcare, Milwaukee, Wisconsin, USA) or the Biograph Vision scanner (Siemens Medical Solutions, Erlangen, Germany). Helical low dose, non-contrast CT images (tube voltage 100 kVp, current intensity 62 mAs, slice thickness 3.75 mm) were obtained for attenuation correction, similar to other groups [18, 19]. PET images were acquired in three-dimensional mode and reconstructed to 128 × 128 image matrices using an iterative algorithm. Images were sent to our institution’s picture archiving and communications system (Sectra PACS, Sectra Medical, Linkoping, Sweden).

### Image Analyses

A fellowship-trained neuroradiologist with 5 years of experience (who was blinded to the HER2 status of the breast cancer brain metastases) placed an approximately 50 mm^2^ circular region of interest (ROI) on the most intensely ^18^F-FDG-avid part of each lesion. A fellowship trained neuroradiologist with 20 years of experience (who was also blinded to the HER2 status of the breast cancer brain metastases) reviewed each ROI for appropriate placement. The maximum standardized uptake value (SUVmax) within each ROI was recorded. An approximately 50 mm^2^ circular ROI was placed in the contralateral normal-appearing white matter as a standard internal reference. The SUVmax ratio was obtained for each lesion by dividing the SUVmax of the lesion by the SUVmax of the contralateral normal-appearing white matter. Each patient in the study cohort also had a preoperative contrast-enhanced brain MRI. The maximal lesion diameter on axial contrast-enhanced images from the preoperative brain MRI was measured to obtain the size of each lesion.

### HER2 Status Assessment

Immunohistochemistry (IHC) and fluorescent in situ hybridization (FISH) were performed to determine the HER2 status of the resected/biopsied brain specimens. The FDA-approved Ventana PATHWAY 4B5 clone (Ventana, Tucson, Arizona, USA) was utilized for IHC analyses. Subspecialty-trained breast pathologists interpreted the IHC analyses according to the 2018 American Society of Clinical Oncology (ASCO)/College of American Pathologists (CAP) practice guidelines [20]. The PathVysion HER2 DNA Probe Kit (Abbott Laboratories, Abbott Park, Illinois, USA) was used for FISH analyses for the detection of HER2 gene amplification. Board-certified cytogeneticists interpreted the FISH analyses according to the 2018 ASCO/CAP practice guidelines. Formalin-fixed, paraffin-embedded tissue sections of the resected or biopsied brain metastases were used for all HER2 tests. A brain metastasis was classified as HER2-positive if the brain metastasis met the criteria for HER2 positivity in the 2018 ASCO/CAP practice guidelines by either FISH or IHC.

All 14 breast cancer brain metastases in our study cohort underwent IHC analyses. Twelve of the 14 breast cancer brain metastases in our cohort underwent FISH analyses. The results of the HER2 IHC and FISH analyses were obtained from the pathology reports in our institution’s electronic medical record. Estrogen receptor (ER) and progesterone receptor (PR) status and tumor grade were also obtained from the pathology reports in our institution’s electronic medical record. For 10 of the 14 breast cancer brain metastases in our cohort, the tumor grade of the breast cancer brain metastasis was specified in the pathology report. For the other 4 breast cancer brain metastases in our study cohort, tumor grade was not specified in the pathology report. In these cases, tumor grade was obtained from the pathology report for the primary breast cancer.

### Statistical Analyses

Mann–Whitney *U* tests were utilized to compare the SUVmax ratio of HER2-positive lesions and HER2-negative lesions. Mann–Whitney *U* and Chi-squared tests were utilized to compare the lesion size and location distribution, as well as the ER and PR status, of the HER2-positive and HER2-negative breast cancer brain metastases. Differences with *p*-values less than 0.05 were considered statistically significant. SPSS 27 (IBM Corp, Armonk, NY, USA) was used to perform the statistical analyses. To account for potential clustering effects, the data were also analyzed including only one lesion per patient. To account for potential effects from scanner differences, the data were analyzed including only lesions that were imaged on the GE Discovery STE scanner. To account for potential effects from differences in tumor grade, the data were analyzed including only grade 3 lesions.

## Results

### HER2-Positive and HER2-Negative Breast Cancer Brain Metastases

Baseline characteristics for the breast cancer brain metastases in our study cohort are shown in Table 1. The mean patient age at diagnosis was 59 years (range: 43–76 years). The mean time from PET/CT imaging to resection or biopsy was 47 days (range: 7–181 days). Of the 14 total breast cancer brain metastases in our study cohort, 10 of the lesions were HER2-positive, and 4 of the lesions were HER2-negative. The mean lesion size was 3.2 cm for HER2-positive breast cancer brain metastases and 3.5 cm for HER2-negative breast cancer brain metastases; this difference was not statistically significant (*U* = 17.00, *p* = 0.73). The location distribution was not significantly different between the HER2-positive and HER2-negative breast cancer brain metastases (*χ*^2^ = 3.38, *p* = 0.34). The ER status (*χ*^2^ = 0.53, *p* = 0.47) and PR status (*χ*^2^ = 1.26, *p* = 0.26) were not significantly different between the HER2-positive and HER2-negative breast cancer brain metastases. The majority of the lesions in our study cohort had a high tumor grade (13/14). Eleven of the lesions were imaged on the GE Discovery STE scanner, while the remaining 3 lesions were imaged on the Siemens Biograph Vision scanner.

**Table 1:** Characteristics of HER2-positive and HER2-negative breast cancer brain metastases

Characteristic	All (*n* = 14 lesions, 12 patients)	HER2-positive (*n* = 10 lesions, 8 patients)	HER2-negative (*n* = 4 lesions, 4 patients)
Mean age in years*	59 (43–76)	56 (44–76)	65 (43–76)
Method of specimen acquisition			
Resection	7 (50)	7 (70)	0 (0)
Biopsy	7 (50)	3 (30)	4 (100)
Lesion size in cm*	3.3 (1.8–5.2)	3.2 (1.8–4.8)	3.5 (1.9–5.2)
Brain location			
Frontal	3 (21)	1 (10)	2 (50)
Temporal	3 (21)	2 (20)	1 (25)
Parietal	2 (14)	2 (20)	0 (0)
Occipital	0 (0)	0 (0)	0 (0)
Cerebellum	6 (43)	5 (50)	1 (25)
HER2 status by IHC			
Positive	10 (71)	10 (100)	0 (0)
Negative	2 (14)	0 (0)	2 (50)
Equivocal	2 (14)	0 (0)	2 (50)
HER2 status by FISH			
Positive	9 (64)	9 (90)	0 (0)
Negative	3 (21)	0 (0)	3 (75)
Not available	2 (14)	1 (10)	1 (25)
ER status by IHC			
Positive	12 (86)	9 (90)	3 (75)
Negative	2 (14)	1 (10)	1 (25)
PR status by IHC			
Positive	4 (29)	2 (20)	2 (50)
Negative	10 (71)	8 (80)	2 (50)
Tumor grade			
Grade 2	1 (7)	0 (0)	1 (25)
Grade 3	13 (93)	10 (100)	3 (75)

Unless otherwise indicated, data are numbers of lesions, with percentages in parentheses

^*^Data in parentheses are the ranges

*HER2*, human epidermal growth factor receptor 2

*IHC*, immunohistochemistry

*FISH*, fluorescent in situ hybridization

*ER*, estrogen receptor

*PR*, progesterone receptor

### SUVmax Ratio

**Fig. 1.**
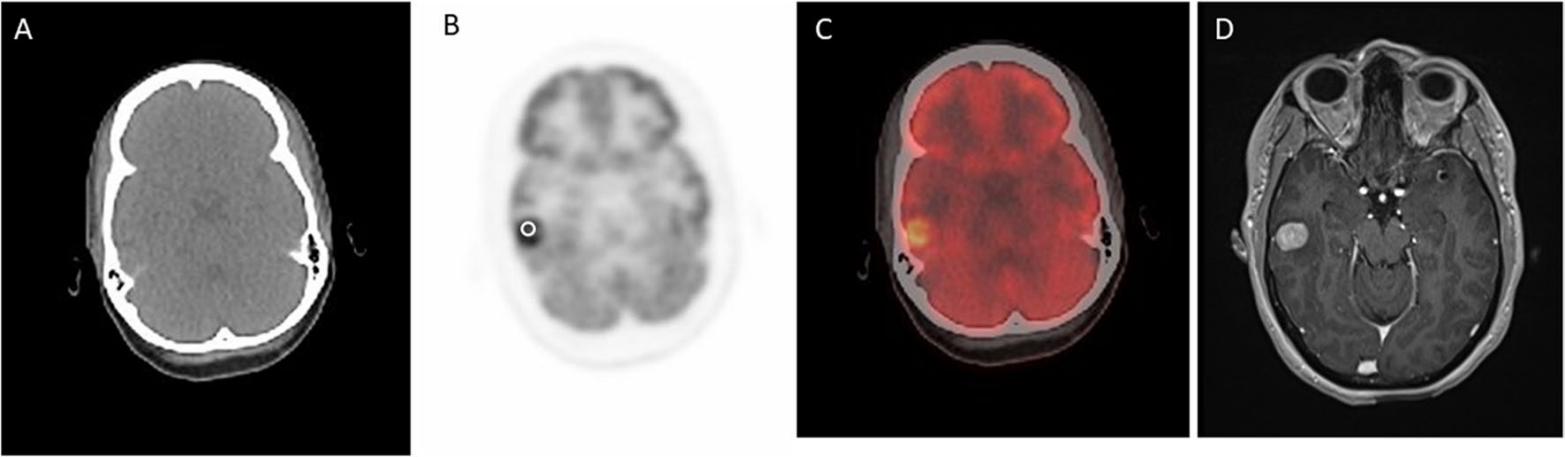
^18^F-FDG PET/CT and contrast-enhanced MRI images of a HER2-positive breast cancer metastasis. Non-contrast CT (**A**), ^18^F-FDG PET (**B**), fused ^18^F-FDG PET and CT (**C**), and axial T1-weighted post-contrast (**D**) images of a HER2-positive breast cancer brain metastasis in a 44-year-old woman. A representative region of interest is shown in white. This lesion had an SUVmax ratio of 4.99. ^18^F-FDG = ^18^F-fluorodeoxyglucose. SUVmax = maximum standardized uptake value

**Fig. 2.**
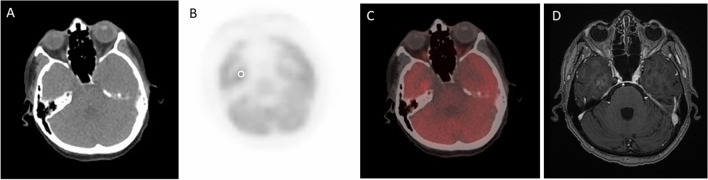
^18^F-FDG PET/CT and contrast-enhanced MRI images of a HER2-negative breast cancer metastasis. Non-contrast CT (**A**), ^18^F-FDG PET (**B**), fused ^18^F-FDG PET and CT (**C**), and axial T1-weighted post-contrast (**D**) images of a HER2-negative breast cancer brain metastasis in a 68-year-old woman. A representative region of interest is shown in white. This lesion had an SUVmax ratio of 2.19. ^18^F-FDG = ^18^F-fluorodeoxyglucose. SUVmax = maximum standardized uptake value

**Fig. 3:**
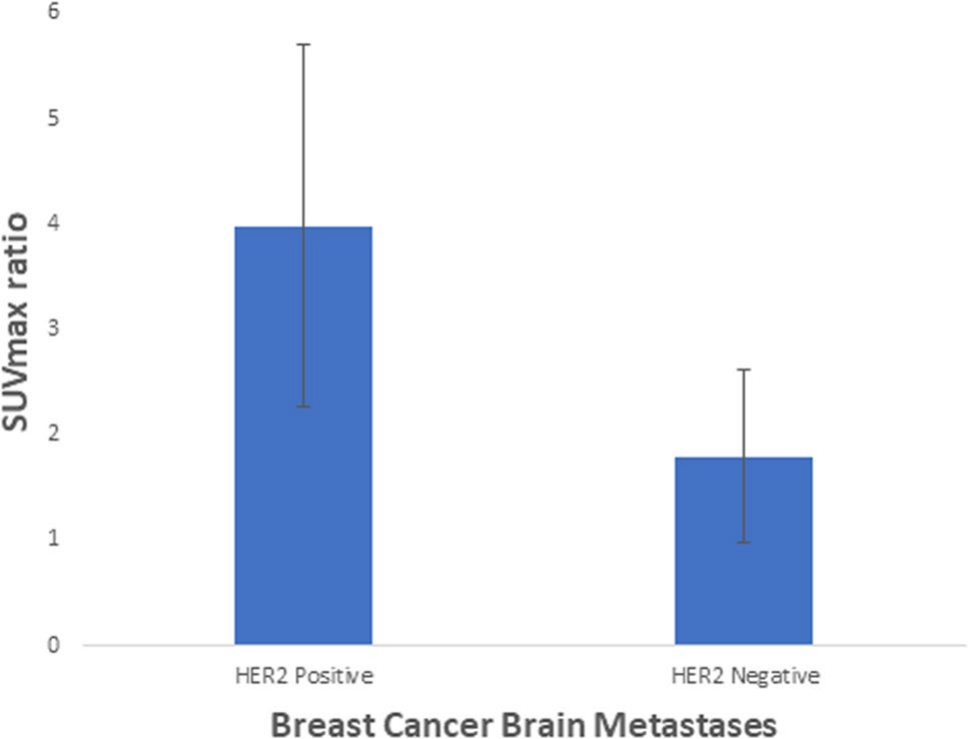
SUVmax ratio of HER2-positive and HER2-negative breast cancer brain metastases. Data points are SUVmax ratio values. Error bars are 95% confidence intervals. SUVmax = maximum standardized uptake value. HER2 = human epidermal growth factor receptor 2

The SUVmax ratio of HER2-positive breast cancer brain metastases was significantly greater than the SUVmax ratio of HER2-negative breast cancer brain metastases (3.98 vs 1.79, *U* = 38.00, *p* = 0.008), as presented in Figs. 1, 2, 3. The SUVmax of HER2-positive breast cancer brain metastases was greater than the SUVmax of HER2-negative breast cancer brain metastases, although this difference was not statistically significant (14.82 vs 7.88, *U* = 32.50, *p* = 0.076). The SUVmax of the contralateral normal-appearing white matter was not significantly different between HER2-positive and HER2-negative breast cancer brain metastases (3.82 vs, 4.48, *U* = 9.50, *p* = 0.142).

To account for potential clustering effects, the data were also analyzed including only one lesion per patient. The results were similar. The SUVmax ratio of HER2-positive lesions was significantly greater than the SUVmax ratio of the HER2-negative lesions (4.31 vs 1.78, *U* = 31.00, *p* = 0.008).

To account for potential effects from scanner differences, the data were analyzed including only lesions that were imaged on the GE Discovery STE scanner. The results were similar. The SUVmax ratio of HER2-positive lesions was significantly greater than the SUVmax ratio of HER2-negative lesions (4.07 vs 1.92, *U* = 22.00, *p* = 0.048).

To account for potential effects from differences in tumor grade, the data were analyzed including only grade 3 lesions. The results were similar. The SUVmax ratio of HER2-positive lesions was significantly greater than the SUVmax ratio of HER2-negative lesions (3.98 vs 1.63, *U* = 30.00, *p* = 0.007).

## Discussion

We found that the SUVmax ratio of HER2-positive breast cancer brain metastases was significantly greater than the SUVmax ratio of HER2-negative breast cancer brain metastases. To our knowledge, our study is the first to demonstrate an association between uptake on ^18^F-FDG PET and the HER2 status of breast cancer brain metastases.

Survival outcomes for patients with HER2-positive metastatic breast cancer have significantly improved due to the development of effective HER2-directed therapies. These therapies have converted what once was an extremely aggressive cancer into a chronic disease. With HER2-directed agents, approximately 38% of patients with HER2-positive metastatic breast cancer are still alive eight years after the development of metastatic disease [21]. Patients with HER2-positive metastatic breast cancer are now living longer, and greater than 50% of women with HER2-positive metastatic breast cancer will develop brain metastases over the course of their disease. Fortunately, these new HER2-directed agents have shown great efficacy in treating brain metastases and thus can delay the need for whole brain irradiation and its accompanying toxicity (most notably cognitive impairment) [2, 22–24]. However, the current assessment of the HER2 status of brain metastases requires pathologic tissue sampling for either IHC or FISH analyses. Because it is often not feasible nor practical to biopsy every CNS lesion, a non-invasive imaging biomarker for HER2 status in breast cancer brain metastases is clearly needed and may help to guide individualized therapy.

The mechanism for the association of ^18^F-FDG PET uptake and HER2 status is not yet known. However, we suspect that this association may be related to HER2-driven glucose uptake and metabolism [16]. HER2 overexpression has been shown to trigger a signal transduction cascade involving dihydroceramide desaturase 1 (DES1) that promotes increased glucose uptake and reprogramming of metabolic pathways [25]. HER2 overexpression in breast cancer cell lines promotes aerobic glycolysis and increased glucose consumption [17].

There are several potential limitations to our study. First, this was a small, proof-of-concept, exploratory study assessing the association between ^18^F-FDG PET uptake and HER2 status. Our findings should be validated in a large prospective trial. The size of our study cohort is partially due to the fact that we included only histologically proven breast cancer brain metastases with HER2 analyses of the resected or biopsied brain specimens. In contradistinction to many of the published studies investigating imaging biomarkers of HER2 status, which have correlated brain MRI imaging parameters with the HER2 status of the primary breast cancer [26–29], our study correlated ^18^F-FDG PET uptake with the HER2 status of the breast cancer brain metastases. This is a crucial distinction, as there is discordance between the HER2 status of the breast cancer brain metastasis and the HER2 status of the primary breast cancer in 10–15% of patients [14, 15]. Second, due to the retrospective nature of our study, all of the brain metastases were not imaged with the same PET/CT scanner. Nevertheless, most of the lesions (11 of 14) were imaged on the GE Discovery STE scanner, while the remaining 3 lesions were imaged on the Siemens Biograph Vision scanner. When the data were analyzed including only lesions imaged on the GE Discovery STE scanner, the results were similar. In addition, in our analyses, SUVmax values were normalized to a standard internal control, contralateral normal-appearing white matter. Third, we did not have follow-up ^18^F-FDG PET imaging for the breast cancer brain metastases in our study cohort. As a result, we could not investigate whether uptake on ^18^F-FDG PET could be utilized longitudinally to evaluate the HER2 status of breast cancer brain metastases.

Several investigators have evaluated MRI biomarkers to determine the HER2 status of breast cancer brain metastases. The intensity of enhancement on contrast-enhanced brain MRI has been shown to have an association with the HER2 status of breast cancer brain metastases [30]. In addition, lesion contour and lesion composition on brain MRI have been shown to be associated with HER2 status [31]. The relative cerebral blood volume from dynamic susceptibility contrast-enhanced perfusion brain MRI has been shown to be associated with HER2 status [32]. Because of the small size of our study cohort, we chose to focus on the difference in ^18^F-FDG PET uptake between HER2-positive and HER2-negative breast cancer brain metastases. Future large prospective trials should evaluate the effectiveness of combining ^18^F-FDG PET uptake and MRI features in differentiating HER2-positive and HER2-negative breast cancer brain metastases.

While breast cancer commonly metastasizes to other organs (such as the lungs and bones), in addition to the brain, our study focused on the association between ^18^F-FDG PET uptake and the HER2 status of breast cancer brain metastases because patients with HER2-positive breast cancer have a greater likelihood of developing brain metastases, and determining the HER2 status of breast cancer brain metastases noninvasively has a direct impact on clinical management. New HER2-directed therapies are effective in treating brain metastases and can potentially spare patients from receiving whole brain irradiation and its associated toxicities. In addition, identifying a non-invasive imaging biomarker for HER2 status in breast cancer brain metastases can potentially spare patients from the risks associated with brain biopsies. Future studies should examine the association between ^18^F-FDG PET uptake and the HER2 status of breast cancer lung and bone metastases.

Uptake on ^18^F-FDG PET may assist in differentiating HER2-positive breast cancer brain metastases from HER2-negative lesions, if validated in a large prospective trial. A non-invasive method of identifying the HER2 status of breast cancer brain metastases may help to guide personalized patient care while avoiding the risks of brain biopsy. Furthermore, a baseline ^18^F-FDG PET prior to treatment may be helpful to evaluate new brain metastases and to compare their ^18^F-FDG uptake profiles to prior treated lesions.

## Data Availability

The data are available to qualified investigators upon reasonable request.

## Abbreviations

HER2: Human epidermal growth factor receptor 2
CNS: Central nervous system
^18^F-FDG: ^18^F-fluorodeoxyglucose
ROI: Region of interest
SUVmax: Maximum standardized uptake value
IHC: Immunohistochemistry
FISH: Fluorescent in situ hybridization
ER: Estrogen receptor
PR: Progesterone receptor
ROC: Receiver operating characteristic
